# How to Classify, Teach, and Learn Ophthalmic Eponyms

**DOI:** 10.7759/cureus.18849

**Published:** 2021-10-18

**Authors:** Steven Yale, Halil Tekiner, Eileen S Yale

**Affiliations:** 1 Internal Medicine, University of Central Florida College of Medicine, Orlando, USA; 2 Department of the History of Medicine and Ethics, Erciyes University School of Medicine, Melikigazi, TUR; 3 General Internal Medicine, University of Florida College of Medicine, Gainesville, USA

**Keywords:** student education, syndromes, physical signs, eponyms, ophthalmology

## Abstract

Introduction

There are limited educational studies on effective ways to teach and learn medical eponyms. While there is no consensus on how to best address this issue, developing novel strategies to teach medical eponyms has become critical in many branches of medicine, including ophthalmology.

Materials & Methods

An ophthalmologic eponymic database was created using eight source texts (e.g., books, encyclopedias, and dictionaries) and included the year the eponym was introduced, related name, nationality, specialty, and the eponym’s description. PubMed database with a Medical Subject Headings (MeSH) keyword for “eponym” and “eye” and “ophthalmology” and a Google search for a combination of related keywords was also performed. A careful biographical search was conducted for each name in the second phase to obtain further biographical details. Inclusion criteria for eponyms in the dataset were: i) named after at least one person, ii) identified as a specific medical term in the literature, iii) related to any field of medicine. Names derived from art, history, mythology, patient, family, chemistry, botany (or other fields outside of medicine) were excluded. The three authors independently screened to eliminate duplicated names and ensure eligible names met inclusion and exclusion criteria.

Results

A total of 1,257 unique ophthalmologic eponyms representing 8.8% of 14,332 medical eponyms were identified. Three-hundred fifty-one of 743 (47.2%) eponyms were named after ophthalmologists representing 36 countries. The United States of America and Germany comprised the largest fraction of nationalities (40.2%), not necessarily representing their birthplace. Signs, syndromes, and diseases composed the largest category (45.8%) of eponymous ophthalmologic names.

Discussion

The current volume of eponymous names impedes the ability of a learner to retain this information. Classifying eponyms based on form, intention, or function, provides a more refined method for placing eponyms in their respective categories. Teaching eponyms by enumerating their historical content, demonstrating the correct performance of the eponym, assessing the technique, and providing feedback, affords the learner a more fruitful and meaningful learning experience. Understanding the context of the signs, syndrome, or techniques further allows the learner to gain insights into the clinical application of eponyms in diagnostic decision-making.

Conclusion

The teaching model proposed incorporates key aspects that may facilitate retention and recall of the eponymous name. The model includes imparting historical knowledge about the person who described the sign, technique, or process; demonstrating the correct procedure as originally reported; and coaching to ensure that the appropriate skill is mastered. Before abandoning eponyms, it is first necessary to understand their efficacy, effectiveness, usefulness, and role in clinical medicine.

## Introduction

Eponyms provide a rich cultural perspective to the literature and remind us about our historical medical heritage. Despite this, the routine use of eponyms remains controversial, with not all physicians embracing their widespread use [1-3]. Unfortunately, ophthalmologic eponyms have not been well studied, and there is a paucity of information available for only a few eponyms in clinical medicine [4,5]. Before abandoning eponyms, it is incumbent that we further study them in order to determine their reliability, validity, and applicability in clinical practice. Eponyms found to be valid should be further classified, based on expert advice within the various subspecialties, to facilitate teaching and learning. This paper aims to formulate and propose a teaching model that provides a meaningful way for classifying and teaching ophthalmological eponyms. We recognize gaps in our knowledge about how to teach eponyms as there are limited studies available. Furthermore, the principles applied to the ophthalmologic literature are also applicable to other specialties of medicine.

## Materials and methods

Data were obtained from research utilizing primarily eight sources representing medical or eponymic books, dictionaries, encyclopedias, indexes, medical periodicals, and one online source. The following criteria were considered in selecting eponyms: i) the eponym should be named after at least one person, ii) the eponym should be identified as a specific medical term in the literature, and iii) the eponyms should be related to any field of medicine. Names derived from art, history, mythology, patient or family names were excluded from the list identified. Moreover, eponymic names related to chemistry, biology, botany (or other fields outside of medicine) were also excluded.

In the first phase, an eponymic database was created. The eight sources were scanned by one of the authors (Halil Tekiner), identifying names, year introduced, related name, nationality and specialty, and the eponym description. The PubMed database was searched with a Medical Subject Headings (MeSH) keyword for “eponym” and “eye” and “ophthalmology” in humans published from inception to June 1, 2021. A Google search for a combination of related keywords was also performed to find any other names not appearing in text sources. A biographical search was conducted for each name in the second phase to obtain further biographical details. Obituaries were extremely helpful in this regard. For those names whose gender was not apparent, a photographic search was also conducted. The three authors independently screened to eliminate duplicated names and ensure eligible names met inclusion and exclusion criteria.

This type of research has its limitations. Despite our efforts, it is inevitable to have several names missed in this list or the derivation undetermined, especially those known only with their surname or the initial letters of their names. Therefore we cannot claim this list is precisely complete in its current form.

## Results

We identified and classified 1,257 (8.8%) unique ophthalmologic eponyms representing 14,332 total eponyms in the eponymous literature [6-13]. We created eight categories within this classification schema as this would limit the number of categories making it easier to manage and learn. Each eponym was assigned to a single category to avoid overlap, even though some fit into more than one. For example, Westphal pupillary reflex is both a clinical and physiologic phenomenon but was assigned to the clinical one, as it is more relevant to a practicing physician. Categories were arranged based on common themes. The category algorithm, classification, formula, theory, and law all involve problem-solving techniques. Clinical symptoms are objective or subjective findings or phenomena. Pathologic findings were grouped with clinical symptoms as they represent the histopathologic component to an objective or subjective condition. The words device, instrument, product, and supply are in reference to a larger category named tools, as a device may be an instrument or equipment, and an instrument is a device. With this exception, all the remaining eponyms received one qualifier within a category. 

Signs, syndromes, and diseases represented the largest category at 576 (45.8%), followed by those including devices, instruments, medical products and supplies at 260 (20.7%), clinical/pathological aspects of disease at 185 (14.7%), techniques at 127 (10.1%), and other subcategories (Table 1).

**Table 1:** Classification of ophthalmologic eponyms

Classification	Frequency of unique eponyms
Algorithm, classification, formula, theory, law	6
Anatomic or physiologic	57
Clinical symptoms or pathologic findings	185
Device, instrument, product, supply (tool)	260
Infectious agents	11
Sign, syndrome, or disease	576
Technique	127
Test or treatment	35

Named after 743 professionals (including 351 ophthalmologists) from 36 countries, the vast majority (97.9%) of these are names honored men, reflecting the preponderance of men in this specialty before the mid-twentieth century. Interestingly, the nationality of these individuals, not necessarily the country from which the eponym was written, were American (20.9%), German (19.3%), and Austrian (10.2%); followed by French (9.8%), Swiss (7.6%), and English (7.1%) among many others.

## Discussion

Eponyms used in the context of clinical medicine are honorific terms bestowed to an individual(s) who identified or discovered a disease, sign, symptom, syndrome, test, or finding. They may also represent a designation of an anatomical structure, devices, procedures or techniques, views or phenomena, treatments, classifications or indexes, prediction rules, laws or principles, or algorithms. Eponymic signs and findings are rarely pathognomonic by themselves. When used in combination with other symptoms, signs, and physical findings, it assists in diagnosis. Their use should be strictly reserved to honor individuals whose contribution(s) embody the rich tradition of the art of medicine for centuries.

There are drawbacks to the use of medical eponyms, which have led to reservations regarding their use. Limitations with eponyms include inaccessibility of the original publication or presentation because it was written in a different language or presented at a conference or in a monograph, historical misinformation, lack of attribution of all authors, naming (e.g., misspelled, a middle name used, multiple renditions of surname, and the same name which may have different meanings), and transcription challenges from non-Latin scripts such as Arabic, Greek, and Russian or sometimes from umlaut letters (ä, ö, ü) in the extended Latin alphabet. Other issues regarding eponyms are the routine use by authors of the possessive form of the name and inclusion within the literature of eponymic names derived from individuals who committed atrocities against humanity or bore racist or antisemitic remarks. The latter has improved as of more recent times as there has been increased awareness and confirmation of these claims. As a result, diseases were renamed and replaced with more descriptive terms (e.g., Wenger granulomatosis to granulomatous with polyangiitis or Reiter syndrome to reactive arthritis). The possessive form of the eponymous name continues to be used in the literature even though its use should be restricted to cases whereby the person had the sign, disease, or syndrome for which they described. The most highly recognized example of where the possessive form is an acceptable designation is Trousseau's sign named for Armand Trousseau (1801-1867), who developed phlebitis occurring in association with a gastric tumor [14].

It is unrealistic to think that hundreds of ophthalmologic eponymic names could be taught, processed, remembered, and recalled. This is compounded in that many eponymous names having more than one designation in the same category. Furthermore, it may not be recognized that an eponym may also be named for both a father and son, as in Sturge-Weber syndrome or von Hippel-Lindau disease. Several papers recommend standardizing terminology when naming new and existing morphological abnormalities and new diseases that can also be applied when classifying ophthalmologic eponyms [15-17]. This serves as the basis for assisting in identifying the framework for naming eponyms. The critical designations to consider are that eponyms be limited to one proper name and no more than three names with the last name(s) used, and authorship preferably limited to include those in the first three positions of the manuscript or monographs. Initials and acronyms [(e.g., acute retinal necrosis (ARN), congenital hypertrophy of the retinal pigment epithelium (CHRPE), and acute posterior multifocal placoid pigment epitheliopathy (APMPPE)] should be avoided. Eponyms that are currently obsolete (e.g., Donder glaucoma, Donder ring, Doyne honeycomb choroidopathy, Filatov operation) should be excluded from ophthalmologic terminology.

Despite these limitations, which apropos constitute a small portion of the eponymous corpus, we believe that eponyms should be retained and further studied unless there is compelling evidence to the contrary. We concur with the words of Sir Gordon Gordon-Taylor (1878-1960), a pioneering British surgeon and past President of the Royal Society of Medicine, “This may sound antiquated, but it goes against me to sacrifice names which for centuries have proved to be good and useful. The honorable names of our science are thereby fixed in the memory of posterity, and through them, there is awakened in the student a certain historical interest which stimulates him to further investigation" [18].

The field of ophthalmology is replete with eponyms that have been assigned to nearly every conceivable structure within the eye or as part of a systemic process that secondarily involves the eye. Interest in eponyms is likely to grow in recent years, with many journals publishing articles on ophthalmologic eponyms such as Eales disease and Sjögren syndrome or eponyms named to honor female ophthalmologists [19-22].

Clarifications regarding eponyms and their clinical applications can only come about through further studies that examine their validity, reliability, and reproducibility. Physicians make cognitive errors in clinical decision-making that involve biases and heuristic shortcuts. This may be further perpetuated based on anchoring and premature closure, which leads physicians astray from identifying the correct diagnosis. Other errors are linguistic, based on a physician's lack of knowledge regarding the meaning of a structure, process, disease, symptom, syndrome, or sign. These failures include physician's inability to distinguish Bell (Charles Bell, 1774-1842) palsy as idiopathic facial nerve paralysis from other known causes of peripheral facial nerve paralysis, or that William John Adie (1886-1935) described the findings of areflexia found predominantly in women with a known tonic pupil and thus constitutes a syndrome (Adie syndrome), a constellation of signs, symptoms, and findings, "All the evidence seems to me to support the notion that the tonic convergence reaction in pupils apparently inactive to light is a thing apart. The peculiar extra-ocular phenomena (symptomless areflexia) that are frequently associated with it also suggests that we are confronted by a unique condition" [22]. Therefore, it is incumbent that educators teach these principles to learners to avoid perpetuating the same errors. The question is, what the best method for teaching eponyms is?

Disease, syndromes, and signs will continue to become better defined as they are studied, leading to, in some cases, a more refined and specific disease classification. Eponymically named signs and syndromes by themselves lack sufficient specificity alone for diagnostic purposes. In most cases, they represent only the anatomic (e.g., Leber venous plexus), physiologic (Donder law), or pathologic (e.g., Fuch adenoma) expression of the disease or algorithm (e.g., Helvacioglu reproducibility index), classification (e.g., Mann), theory (e.g., Elschnig), or formula (e.g., Reuss). These eponymic signs, syndromes, findings, devices, and techniques, when used in some cases in combination with other more sophisticated diagnostic tests, assist in better understanding, sorting out, and sorting through, the various disease processes.

Classifying ophthalmologic eponyms based on a mechanistic approach assists the reader to understand better their representation (e.g., anatomic, pathologic, physiologic) and purpose (e.g., test, technique, algorithm, classification, instrument, or device, or operative technique), as shown in Table 2. 

**Table 2:** Common representative ophthalmologic eponym names

Name	Eponym	Classification
Adie, William John (1886-1935)	Adie pupil	clinical symptoms
Adie, William John (1886-1935)	Adie syndrome	syndrome
Adie, William John (1886-1935)	Adie-Critchley syndrome	syndrome
Amsler, Marc (1891-1968)	Amsler chart	tool
Amsler, Marc (1891-1968)	Amsler chart marker	tool
Amsler, Marc (1891-1968)	Amsler corneal graft operation	technique
Amsler, Marc (1891-1968)	Amsler grid	tool
Amsler, Marc (1891-1968)	Amsler needle	tool
Amsler, Marc (1891-1968)	Amsler scleral marker	tool
Amsler, Marc (1891-1968)	Amsler test	test
Amsler, Marc (1891-1968)	Amsler-Verry sign	sign
Arlt, Carl Ferdinand Ritter von (1812-1887)	Arlt eyelid repair	technique
Arlt, Carl Ferdinand Ritter von (1812-1887)	Arlt fenestrated lens scoop	tool
Arlt, Carl Ferdinand Ritter von (1812-1887)	Arlt lens loupe	tool
Arlt, Carl Ferdinand Ritter von (1812-1887)	Arlt line	pathologic findings
Arlt, Carl Ferdinand Ritter von (1812-1887)	Arlt operation	technique
Arlt, Carl Ferdinand Ritter von (1812-1887)	Arlt pterygium excision	technique
Arlt, Carl Ferdinand Ritter von (1812-1887)	Arlt recess	anatomic
Arlt, Carl Ferdinand Ritter von (1812-1887)	Arlt sinus	anatomic
Arlt, Carl Ferdinand Ritter von (1812-1887)	Arlt sutures	tool
Arlt, Carl Ferdinand Ritter von (1812-1887)	Arlt syndrome	syndrome
Arlt, Carl Ferdinand Ritter von (1812-1887)	Arlt trachoma	pathologic findings
Arlt, Carl Ferdinand Ritter von (1812-1887)	Arlt triangle	pathologic findings
Axenfeld, Karl Theodor Paul Polykarpus (1867-1930)	Axenfeld calcareous degeneration	pathologic findings
Axenfeld, Karl Theodor Paul Polykarpus (1867-1930)	Axenfeld syndrome	syndrome
Behçet, Hulusi (1889–1948)	Behçet disease	disease
Behçet, Hulusi (1889–1948)	Behçet syndrome	syndrome
Behr, Carl Julius Peter (1874-1943)	Behr abduction phenomenon	clinical symptoms
Behr, Carl Julius Peter (1874-1943)	Behr disease	disease
Behr, Carl Julius Peter (1874-1943)	Behr sign	sign
Behr, Carl Julius Peter (1874-1943)	Behr syndrome	syndrome
Bell, Sir Charles (1774-1842)	Bell law	law
Bell, Sir Charles (1774-1842)	Bell nerve	anatomic
Bell, Sir Charles (1774-1842)	Bell palsy	clinical symptoms
Bell, Sir Charles (1774-1842)	Bell paralysis	clinical symptoms
Bell, Sir Charles (1774-1842)	Bell phenomena	clinical symptoms
Best, Franz (1878-1920)	Best carmine stain	test
Best, Franz (1878-1920)	Best disease	disease
Best, Franz (1878-1920)	Best macular degeneration	pathologic findings
Bielschowsky, Alfred (1871-1940)	Bielschowsky disease	disease
Bielschowsky, Alfred (1871-1940)	Bielschowsky head tilt test	test
Bielschowsky, Alfred (1871-1940)	Bielschowsky method	technique
Bielschowsky, Alfred (1871-1940)	Bielschowsky phenomenon	clinical symptoms
Bielschowsky, Alfred (1871-1940)	Bielschowsky sign	sign
Bielschowsky, Alfred (1871-1940)	Bielschowsky squint	clinical symptoms
Bielschowsky, Alfred (1871-1940)	Bielschowsky stain	test
Bielschowsky, Alfred (1871-1940)	Bielschowsky syndrome	syndrome
Bielschowsky, Alfred (1871-1940)	Bielschowsky-Dollinger syndrome	syndrome
Bjerrum, Jannik Petersen (1851-1926)	Bjerrum scotoma	pathologic findings
Bjerrum, Jannik Petersen (1851-1926)	Bjerrum scotometer	tool
Bjerrum, Jannik Petersen (1851-1926)	Bjerrum screen	tool
Bjerrum, Jannik Petersen (1851-1926)	Bjerrum sign	sign
Bowman, Sir William (1816-1892)	Bowman eye knife	tool
Bowman, Sir William (1816-1892)	Bowman iris needle	tool
Bowman, Sir William (1816-1892)	Bowman iris scissors	tool
Bowman, Sir William (1816-1892)	Bowman lacrimal dilator	tool
Bowman, Sir William (1816-1892)	Bowman lacrimal probe	tool
Bowman, Sir William (1816-1892)	Bowman lamellae of cornea	anatomic
Bowman, Sir William (1816-1892)	Bowman membrane	anatomic
Bowman, Sir William (1816-1892)	Bowman probe	tool
Bowman, Sir William (1816-1892)	Bowman strabismus scissors	tool
Bruch, Karl Wilhelm Ludwig (1819-1884)	Bruch glands	pathologic findings
Bruch, Karl Wilhelm Ludwig (1819-1884)	Bruch membrane	anatomic
Cloquet, Jules Germain (1790-1883)	Cloquet canal	anatomic
Cloquet, Jules Germain (1790-1883)	Cloquet canal remnants	anatomic
Cloquet, Jules Germain (1790-1883)	Cloquet space	anatomic
Coats, George (1876-1915)	Coats disease	disease
Coats, George (1876-1915)	Coats ring	pathologic findings
Coats, George (1876-1915)	Coats syndrome	syndrome
Cogan, David Glendenning (1908-1993)	Cogan microcystic dystrophy	pathologic findings
Cogan, David Glendenning (1908-1993)	Cogan sign	sign
Cogan, David Glendenning (1908-1993)	Cogan syndrome	syndrome
Cogan, David Glendenning (1908-1993)	Cogan-Reese disease	disease
Dalrymple, John (1803-1852)	Dalrymple disease	disease
Dalrymple, John (1803-1852)	Dalrymple sign	sign
Doyne, Robert Walter (1857-1916)	Doyne choroiditis	pathologic findings
Doyne, Robert Walter (1857-1916)	Doyne guttate iritis	pathologic findings
Doyne, Robert Walter (1857-1916)	Doyne honeycomb choroidopathy	pathologic findings
Doyne, Robert Walter (1857-1916)	Doyne operation	technique
Duane, Alexander (1858-1926)	Duane parallax test	test
Duane, Alexander (1858-1926)	Duane retraction syndrome	syndrome
Duane, Alexander (1858-1926)	Duane syndrome	syndrome
Duane, Alexander (1858-1926)	Duane test	test
Eales, Henry (1852-1913)	Eales disease	disease
Edinger, Ludwig (1855-1918)	Edinger-Westphal nucleus	anatomic
Elschnig, Anton (1863-1939)	Elschnig blepharrorrhaphy	technique
Elschnig, Anton (1863-1939)	Elschnig bodies	pathologic findings
Elschnig, Anton (1863-1939)	Elschnig canthorrhaphy	technique
Elschnig, Anton (1863-1939)	Elschnig cataract knife	tool
Elschnig, Anton (1863-1939)	Elschnig conjunctivitis	pathologic findings
Elschnig, Anton (1863-1939)	Elschnig corneal knife	tool
Elschnig, Anton (1863-1939)	Elschnig cyclodialysis spatula	tool
Elschnig, Anton (1863-1939)	Elschnig dissecting knife	tool
Elschnig, Anton (1863-1939)	Elschnig extrusion needle	tool
Elschnig, Anton (1863-1939)	Elschnig eye spoon	tool
Elschnig, Anton (1863-1939)	Elschnig fixation forceps	tool
Elschnig, Anton (1863-1939)	Elschnig iridectomy	technique
Elschnig, Anton (1863-1939)	Elschnig lid retractor	tool
Elschnig, Anton (1863-1939)	Elschnig pearls	pathologic findings
Elschnig, Anton (1863-1939)	Elschnig procedure	technique
Elschnig, Anton (1863-1939)	Elschnig pterygium knife	tool
Elschnig, Anton (1863-1939)	Elschnig refractor	tool
Elschnig, Anton (1863-1939)	Elschnig spots	pathologic findings
Elschnig, Anton (1863-1939)	Elschnig syndrome	syndrome
Elschnig, Anton (1863-1939)	Elschnig theory	theory
Elschnig, Anton (1863-1939)	Elschnig trephine	tool
Fleischer, Bruno Otto (1874-1965)	Fleischer corneal ring	pathologic findings
Fleischer, Bruno Otto (1874-1965)	Fleischer dystrophy	pathologic findings
Fleischer, Bruno Otto (1874-1965)	Fleischer lines	pathologic findings
Fleischer, Bruno Otto (1874-1965)	Fleischer ring	pathologic findings
Fleischer, Bruno Otto (1874-1965)	Fleischer vortex	pathologic findings
Fleischer, Bruno Otto (1874-1965)	Fleischer-Strumpell ring	pathologic findings
François, Émile Jules Marie Joseph (1907-1984)	François cloudy central dystrophy	pathologic findings
François, Émile Jules Marie Joseph (1907-1984)	François dyscelphalic syndrome	syndrome
François, Émile Jules Marie Joseph (1907-1984)	François dystrophy (I)	pathologic findings
François, Émile Jules Marie Joseph (1907-1984)	François dystrophy (II)	pathologic findings
François, Émile Jules Marie Joseph (1907-1984)	François speckled dystrophy	pathologic findings
François, Émile Jules Marie Joseph (1907-1984)	François-Evans syndrome	syndrome
Fuchs, Ernst (1851-1930)	Fuchs adenoma	pathologic findings
Fuchs, Ernst (1851-1930)	Fuchs atrophy	pathologic findings
Fuchs, Ernst (1851-1930)	Fuchs black spots	pathologic findings
Fuchs, Ernst (1851-1930)	Fuchs capsule forceps	tool
Fuchs, Ernst (1851-1930)	Fuchs capsulotomy forceps	tool
Fuchs, Ernst (1851-1930)	Fuchs coloboma	pathologic findings
Fuchs, Ernst (1851-1930)	Fuchs corneal dystrophy	pathologic findings
Fuchs, Ernst (1851-1930)	Fuchs crypt	anatomic
Fuchs, Ernst (1851-1930)	Fuchs dellen	pathologic findings
Fuchs, Ernst (1851-1930)	Fuchs dimples	pathologic findings
Fuchs, Ernst (1851-1930)	Fuchs disease	disease
Fuchs, Ernst (1851-1930)	Fuchs dystrophy	pathologic findings
Fuchs, Ernst (1851-1930)	Fuchs epithelial dystrophy	pathologic findings
Fuchs, Ernst (1851-1930)	Fuchs grid	tool
Fuchs, Ernst (1851-1930)	Fuchs heterochromic cyclitis	pathologic findings
Fuchs, Ernst (1851-1930)	Fuchs keratome	tool
Fuchs, Ernst (1851-1930)	Fuchs lid	pathologic findings
Fuchs, Ernst (1851-1930)	Fuchs phenomenon	clinical symptoms
Fuchs, Ernst (1851-1930)	Fuchs signs	sign
Fuchs, Ernst (1851-1930)	Fuchs spot	pathologic findings
Fuchs, Ernst (1851-1930)	Fuchs stoma	anatomic
Fuchs, Ernst (1851-1930)	Fuchs superficial marginal keratitis	pathologic findings
Fuchs, Ernst (1851-1930)	Fuchs syndrome (I,II)	syndrome
Fuchs, Ernst (1851-1930)	Fuchs two-way eye syringe	tool
Fuchs, Ernst (1851-1930)	Fuchs uveitis	pathologic findings
Fuchs, Ernst (1851-1930)	Fuchs-Kraupa syndrome	syndrome
Goldmann, Hans (1899-1991)**	Goldmann applanation tonometer	tool
Goldmann, Hans (1899-1991)**	Goldmann contact lens prism	tool
Goldmann, Hans (1899-1991)**	Goldmann expressor	tool
Goldmann, Hans (1899-1991)**	Goldmann goniolens	tool
Goldmann, Hans (1899-1991)**	Goldmann macular contact lens	tool
Goldmann, Hans (1899-1991)**	Goldmann multimirror lens implant	tool
Goldmann, Hans (1899-1991)**	Goldmann perimeter	tool
Goldmann, Hans (1899-1991)**	Goldmann serrated knife	tool
Goldmann, Hans (1899-1991)**	Goldmann three-mirror contact lens	tool
Goldmann, Hans (1899-1991)**	Goldmann-Favre disease	disease
Gräfe (Graefe), F.W. Ernst Albrecht von (1828-1870)	Graefe cautery (electrocautery)	tool
Gräfe (Graefe), F.W. Ernst Albrecht von (1828-1870)	Graefe cystotome	tool
Gräfe (Graefe), F.W. Ernst Albrecht von (1828-1870)	Graefe disease	disease
Gräfe (Graefe), F.W. Ernst Albrecht von (1828-1870)	Graefe knife needle	tool
Gräfe (Graefe), F.W. Ernst Albrecht von (1828-1870)	Graefe sign	sign
Graves, Robert James (1796-1853)	Graves ophthalmopathy	clinical symptoms
Graves, Robert James (1796-1853)	Graves orbitopathy	clinical symptoms
Gunn, Robert Marcus (1850-1909)	Gunn dots	anatomic
Gunn, Robert Marcus (1850-1909)	Gunn pupil	clinical symptoms
Gunn, Robert Marcus (1850-1909)	Gunn sign	sign
Haab, Otto (1850-1931)	Haab degeneration	pathologic findings
Haab, Otto (1850-1931)	Haab eye knife	tool
Haab, Otto (1850-1931)	Haab line	pathologic findings
Haab, Otto (1850-1931)	Haab magnet	tool
Haab, Otto (1850-1931)	Haab needle	tool
Haab, Otto (1850-1931)	Haab reflex	physiologic
Haab, Otto (1850-1931)	Haab scleral resection knife	tool
Haab, Otto (1850-1931)	Haab senile macular degeneration	pathologic findings
Haab, Otto (1850-1931)	Haab-Dimmer dystrophy	pathologic findings
Harada, Einosuke (1892-1946)	Harada disease	disease
Harada, Einosuke (1892-1946)	Harada syndrome	syndrome
Henle, Friedrich Gustav Jakob (1809-1885)	Hassle-Henle bodies	pathologic findings
Henle, Friedrich Gustav Jakob (1809-1885)	Henle fiber layer	anatomic
Henle, Friedrich Gustav Jakob (1809-1885)	Henle glands	anatomic
Henle, Friedrich Gustav Jakob (1809-1885)	Henle membrane	anatomic
Henle, Friedrich Gustav Jakob (1809-1885)	Henle nervous layer	anatomic
Hering, Karl Ewald Konstantin (1834-1918)	Hering afterimage	physiologic
Hering, Karl Ewald Konstantin (1834-1918)	Hering test	test
Hering, Karl Ewald Konstantin (1834-1918)	Hering theory of color vision	theory
Hering, Karl Ewald Konstantin (1834-1918)	Hering-Bielschowsky test	test
Hess, Carl von (1863-1923)	Hess capsule iris forceps	tool
Hess, Carl von (1863-1923)	Hess chart	test
Hess, Carl von (1863-1923)	Hess expressor	tool
Hess, Carl von (1863-1923)	Hess eyelid operation	technique
Hess, Carl von (1863-1923)	Hess lens scoop	tool
Hess, Carl von (1863-1923)	Hess lens spoon	tool
Hess, Carl von (1863-1923)	Hess ptosis operation	technique
Hess, Carl von (1863-1923)	Hess screen	test
Hess, Carl von (1863-1923)	Hess test	test
Hippel, Eugen Adolf Arthur von (1867-1939)	Hippel keratoplasty	technique
Hippel, Eugen Adolf Arthur von (1867-1939)	Hippel trephine	tool
Hippel, Eugen Adolf Arthur von (1867-1939)	Lindau-von Hippel syndrome	syndrome
Hippel, Eugen Adolf Arthur von (1867-1939)	von Hippel disease	disease
Hippel, Eugen Adolf Arthur von (1867-1939)	von Hippel ulcer	pathologic findigns
Horner, Johann Friedrich (1831-1886)	Horner hollow chisel	tool
Horner, Johann Friedrich (1831-1886)	Horner pupil	clinical symptoms
Horner, Johann Friedrich (1831-1886)	Horner sign	sign
Horner, Johann Friedrich (1831-1886)	Horner syndrome	syndrome
Horner, Johann Friedrich (1831-1886)	Horner-Trantas dots	pathologic findings
Horner, Johann Friedrich (1831-1886)	Horner-Trantas spots	clinical symptoms
Hutchinson, Sir Jonathan (1828-1913)	Hutchinson facies	clinical symptoms
Hutchinson, Sir Jonathan (1828-1913)	Hutchinson patch	pathologic findings
Hutchinson, Sir Jonathan (1828-1913)	Hutchinson pupil	clinical symptoms
Hutchinson, Sir Jonathan (1828-1913)	Hutchinson sign	sign
Hutchinson, Sir Jonathan (1828-1913)	Hutchinson syndrome	syndrome
Hutchinson, Sir Jonathan (1828-1913)	Hutchinson triad	clinical symptoms
Hutchinson, Sir Jonathan (1828-1913)	Hutchinson tumor	pathologic findings
Knapp, Herman Jakob (1832-1911)**	Knapp cataract knife	tool
Knapp, Herman Jakob (1832-1911)**	Knapp eye speculum	tool
Knapp, Herman Jakob (1832-1911)**	Knapp iris hook	tool
Knapp, Herman Jakob (1832-1911)**	Knapp iris knife needle	tool
Knapp, Herman Jakob (1832-1911)**	Knapp iris repositor	tool
Knapp, Herman Jakob (1832-1911)**	Knapp iris scissors	tool
Knapp, Herman Jakob (1832-1911)**	Knapp iris spatula	tool
Knapp, Herman Jakob (1832-1911)**	Knapp lacrial sac refractor	tool
Knapp, Herman Jakob (1832-1911)**	Knapp lens scoop	tool
Knapp, Herman Jakob (1832-1911)**	Knapp lid operation	technique
Knapp, Herman Jakob (1832-1911)**	Knapp pterygium operation	technique
Knapp, Herman Jakob (1832-1911)**	Knapp streaks	pathologic findings
Knapp, Herman Jakob (1832-1911)**	Knapp striae	pathologic findings
Knapp, Herman Jakob (1832-1911)**	Knapp test	test
Koyanagi, Yosizo (1880-1954)	Vogt-Koyanagi syndrome	syndrome
Krause, Karl Friedrich Theodor (1797-1868)	Krause gland	anatomic
Laurence, John Zachariah (1829-1870)	Laurence-Moon syndrome	syndrome
Le Fort, Léon Clément (1829-1893)	Le Fort fracture	pathologic findings
Leber, Theodor (1840-1917)	Leber cell	pathologic findings
Leber, Theodor (1840-1917)	Leber congenital amaurosis	pathologic findings
Leber, Theodor (1840-1917)	Leber disease	disease
Leber, Theodor (1840-1917)	Leber hereditary optic atrophy	pathologic findings
Leber, Theodor (1840-1917)	Leber idiopathic stellate neuroretinitis	pathologic findings
Leber, Theodor (1840-1917)	Leber miliary aneurysm	pathologic findings
Leber, Theodor (1840-1917)	Leber plexus	anatomic
Leber, Theodor (1840-1917)	Leber retinitis	pathologic findings
Leber, Theodor (1840-1917)	Leber syndrome	syndrome
Leber, Theodor (1840-1917)	Leber venous plexus	anatomic
Lindau, Arvid Wilhelm (1892-1958)	Lindau disease	disease
Maddox, Ernest Edmund (1860-1933)	Maddox prism	tool
Maddox, Ernest Edmund (1860-1933)	Maddox rod	tool
Maddox, Ernest Edmund (1860-1933)	Maddox rod occluder	tool
Maddox, Ernest Edmund (1860-1933)	Maddox rod test	test
Marfan, Antoine Bernard-Jean (1858-1942)	Marfan syndrome	syndrome
Marfan, Antoine Bernard-Jean (1858-1942)	Marfan-Madelung syndrome	syndrome
Mikulicz-Radecki, Johannes (Jan) Anton von (1850-1905)	Mikulicz syndrome	syndrome
Möbius (Moebius), Paul Julius (1853-1907)	Möbius sign	sign
Möbius (Moebius), Paul Julius (1853-1907)	Möbius syndrome	syndrome
Moll, Jacob Antonius (1832-1914)	Moll glands	anatomic
Moon, Robert Charles (1844-1914)	Laurence-Moon syndrome	syndrome
Mooren, Albert (1828-1899)	Mooren ulcer	pathologic findings
Morgagni, Giovanni Battista (1682-1771)	Morgagni cataract	pathologic findings
Müller, Heinrich (1820-1864)	Müller fibres	anatomic
Parinaud, Henri (1844-1905)	Parinaud syndrome	syndrome
Purkinje, Jan Evangelista (1787-1869)	Purkinje afterimage	physiologic
Purkinje, Jan Evangelista (1787-1869)	Purkinje figures	physiologic
Purkinje, Jan Evangelista (1787-1869)	Purkinje images (Purkinje-Sanson images)	physiologic
Purkinje, Jan Evangelista (1787-1869)	Purkinje phenomenon (effect, shift)	physiologic
Purtscher, Othmar (1852-1927)	Purtscher retinopathy (syndrome)	syndrome
Recklinghausen, Friedrich Daniel von (1833-1910)	Recklinghausen disease	disease
Robertson, Douglas Moray Cooper Lamb Argyll (1837-1909)	Argyll-Robertson pupil sign	sign
Robertson, Douglas Moray Cooper Lamb Argyll (1837-1909)	Argyll-Robertson syndrome	syndrome
Sachs, Bernard (Barney) (1858-1944)	Sachs lamp	test
Sachs, Bernard (Barney) (1858-1944)	Tay-Sachs disease	disease
Salzmann, Maximilian (1862-1954)	Salzmann dystrophy	pathologic findings
Salzmann, Maximilian (1862-1954)	Salzmann membrane	anatomic
Sattler, Hubert (1844-1928)	Sattler couche	anatomic
Sattler, Hubert (1844-1928)	Sattler elastic layer	anatomic
Sattler, Hubert (1844-1928)	Sattler glands	anatomic
Sattler, Hubert (1844-1928)	Sattler veil	pathologic findings
Schirmer, Otto W.A. (1864-1917)	Schirmer sign	sign
Schirmer, Otto W.A. (1864-1917)	Schirmer test	test
Schlemm, Friedrich (1795-1858)	Schlemm canal	anatomic
Seidel, Erich (1882-1948)	Seidel scotoma	pathologic findings
Seidel, Erich (1882-1948)	Seidel sign	sign
Seidel, Erich (1882-1948)	Seidel test	test
Sherrington, Sir Charles Scott (1857-1952)	Sherrington law	law
Sjögren, Henrik Samuel Conrad (1899-1986)	Sjögren syndrome (disease)	syndrome
Snellen, Herman (1834-1908)	Snellen chart	test
Snellen, Herman (1834-1908)	Snellen conventional reform implant	technique
Snellen, Herman (1834-1908)	Snellen entropion forceps	tools
Snellen, Herman (1834-1908)	Snellen entropion sutures	tools
Snellen, Herman (1834-1908)	Snellen eye implant	technique
Snellen, Herman (1834-1908)	Snellen fraction	clinical symptoms
Snellen, Herman (1834-1908)	Snellen garden	test
Snellen, Herman (1834-1908)	Snellen letters	test
Snellen, Herman (1834-1908)	Snellen operation	technique
Snellen, Herman (1834-1908)	Snellen reflex	physiologic
Snellen, Herman (1834-1908)	Snellen reform eye	tool
Snellen, Herman (1834-1908)	Snellen reform implant	tool
Snellen, Herman (1834-1908)	Snellen sign	sign
Snellen, Herman (1834-1908)	Snellen soft contact lens	tool
Snellen, Herman (1834-1908)	Snellen suture	tool
Snellen, Herman (1834-1908)	Snellen test	test
Snellen, Herman (1834-1908)	Snellen test types	test
Snellen, Herman (1834-1908)	Snellen vectis	tool
Sömmerring (Soemmerring), Samuel Thomas von (1755-1830)	Sömmerring foramen	anatomic
Sömmerring (Soemmerring), Samuel Thomas von (1755-1830)	Sömmerring ligament	anatomic
Sömmerring (Soemmerring), Samuel Thomas von (1755-1830)	Sömmerring ring cataract	pathologic findings
Sömmerring (Soemmerring), Samuel Thomas von (1755-1830)	Sömmerring spot	anatomic
Stargardt, Karl Bruno (1875-1927)	Stargardt-Behr disease (syndrome)	disease
Sturge, William Allen (1850-1919)	Sturge-Kalischer-Weber syndrome	syndrome
Sturge, William Allen (1850-1919)	Sturge-Weber disease	disease
Sturge, William Allen (1850-1919)	Sturge-Weber syndrome	syndrome
Tay, Warren (1843-1927)	Tay cherry-red spot	pathologic findings
Tay, Warren (1843-1927)	Tay choroiditis	pathologic findings
Tay, Warren (1843-1927)	Tay sign	sign
Tay, Warren (1843-1927)	Tay spot	pathologic findings
Tay, Warren (1843-1927)	Tay syndrome	syndrome
Tay, Warren (1843-1927)	Tay-Sachs disease	disease
Tenon, Jacques-René (1724-1816)	Tenon capsule	anatomic
Tenon, Jacques-René (1724-1816)	Tenon space	anatomic
Terrien, Félix (1872-1940)	Terrien disease	disease
Terrien, Félix (1872-1940)	Terrien marginal degeneration	pathologic findings
Terrien, Félix (1872-1940)	Terrien-Veul syndrome	syndrome
Terson, Albert (1867-1935)	Terson forceps	tool
Terson, Albert (1867-1935)	Terson glands	anatomic
Terson, Albert (1867-1935)	Terson speculum	tool
Terson, Albert (1867-1935)	Terson syndrome (disease)	syndrome
Treacher Collins, Edward (1862-1932)	Treacher Collins syndrome	syndrome
Uhthoff, Wilhelm (1853-1927)	Uhthoff phenomenon	clinical symptoms
Usher, Charles Howard (1865-1942)	Usher syndrome	syndrome
Vogt, Alfred (1879-1943)	Limbal girdle of Vogt	pathologic findings
Vogt, Alfred (1879-1943)	Vogt anterior mosic crocodile shagreen	pathologic findings
Vogt, Alfred (1879-1943)	Vogt striae	pathologic findings
Vogt, Alfred (1879-1943)	Vogt-Koyanagi-Harada (VKH) syndrome	syndrome
Waardenburg, Petrus Johannes (1886-1979)	Waardenburg-Jonkers syndrome	syndrome
Weber, Frederick Parkes (1863-1962)	Rendu-Osler-Weber syndrome	syndrome
Weber, Frederick Parkes (1863-1962)	Sturge-Kalischer-Weber syndrome	syndrome
Westphal, Karl Friedrich Otto (1833-1890)	Strümpell-Westphal disease (pseudosclerosis)	disease
Westphal, Karl Friedrich Otto (1833-1890)	Westphal nucleus	anatomic
Westphal, Karl Friedrich Otto (1833-1890)	Westphal pupillary reflex	clinical symptoms
Westphal, Karl Friedrich Otto (1833-1890)	Westphal-Piltz sign	sign
Wintersteiner, Hugo (1865-1918)	Flexner-Wintersteiner rosettes	pathologic findings
Zinn, Johann Gottfried (1727-1759)	Zinn artery	anatomic
Zinn, Johann Gottfried (1727-1759)	Zinn corona	anatomic
Zinn, Johann Gottfried (1727-1759)	Zinn ligament	anatomic
Zinn, Johann Gottfried (1727-1759)	Zinn membrane	anatomic
Zinn, Johann Gottfried (1727-1759)	Zinn ring	anatomic
Zinn, Johann Gottfried (1727-1759)	Zinn vascular circle	anatomic
Zinn, Johann Gottfried (1727-1759)	Zinn zone (zonule)	anatomic

Eponyms that are studied and deemed most relevant to a particular aspect of ophthalmology (e.g., operative, instruments, clinical, and pathologic) should be identified by a panel of experts, emphasized, and taught.

We propose a learning model that involves teaching historical aspects of the person(s) who described the sign, the signs as originally described, and its application, if available, in medical practice. Teaching history imparts purpose to the eponym and, in some cases, a more in-depth understanding of the process or steps involved in identifying the particular finding. Teaching the finding as described by the author avoids misattribution or communication errors. Lastly, the application of the finding in clinical practice should be covered, incorporating known and evolving techniques and technologies to understand the disease or disease process better.

To the best of our knowledge, only one recent study evaluated a method for teaching eponyms. Viveen et al. assessed knowledge involving ten common eponymous questions before and after a two-day course among 20 orthopedic trauma surgeons in an Arbeitsgemeinschaft für Osteosynthesefragen (Swiss working group for bone fusion issues) advanced trauma course on complex elbow fractures [23]. The eponym questions covered the areas of surgical techniques, fracture types, injury, and pathologic findings. The training involved didactic and cadaveric sessions. In order to prevent bias, no emphasis was placed on the eponyms, and participants were unaware of the nature of the study. The study found that correct answers about the eponym improved in only one question with inter-rater reliability (Kappa score) of 0.31 and 0.37 before and after the course, respectively [23]. Findings from this limited study suggest that this is an ineffective method for teaching eponyms.

We believe that eponyms are interesting to learn, and information about them should be retained and taught using an evidence-based approach. When teaching eponyms, we recommend that the components include a brief historical perspective of the person who described the sign, its original description, and its application in clinical practice. The teacher should explain and, in some cases, demonstrate the proper performance of the eponym and its application in clinical practice. The eponym should be practiced with feedback provided, and in the case of eponyms involving signs, their utility in assisting in diagnosis be emphasized [5,24].

## Conclusions

Teaching and learning eponyms in the context of a historical perspective and, as described by the author, tells a story using a case-based learning applied model approach. This way of learning provides a meaningful way best to understand the application of eponyms in clinical practice. There is a continued impetus to remove eponyms and to substitute them using more descriptive terms. Although we do not favor this approach, we emphasize that eponyms must first be studied before devoting time to this endeavor to determine their effectiveness in clinical medicine. Those eponyms deemed to have utility in clinical practice should be retained and classified using predetermined criteria.
